# Unveiling Vascular Dynamics: Introducing the *Biomedicines* Special Issue on Angiogenesis

**DOI:** 10.3390/biomedicines13122868

**Published:** 2025-11-25

**Authors:** Angela Orecchia, Cristina M. Failla

**Affiliations:** Experimental Immunology Laboratory, IDI-IRCCS (Istituto Dermopatico Dell’Immacolata, Istituto di Ricovero e Cura a Carattere Scientifico), 00167 Rome, Italy

Angiogenesis—the sprouting of new blood vessels from pre-existing ones—has long fascinated biologists and clinicians alike. It is a process that seems almost deceptively simple, yet its impact is vast [1]; without angiogenesis, our tissues could not receive the oxygen and nutrients they require, wounds would never heal, and organs under stress could not adapt [2,3,4]. At the same time, the very same process that rescues ischemic tissues can betray us when we experience disease, fueling tumor growth, sustaining chronic inflammation, and driving pathological remodeling [5]. It is this duality—as a healer and saboteur—that makes angiogenesis such an enduring and compelling subject of research [6].

In this Special Issue, we collate a collection of works that aim to shed new light on both the molecular choreography and the clinical relevance of this vascular phenomenon, with an ambition to gather contributions that do not merely catalog angiogenic factors or pathways, but that also frame angiogenesis within the broader story of health, disease, and therapeutic innovation.

Interest in angiogenesis is hardly new; since Judah Folkman first proposed that tumors depend on their own blood supply [7], the biomedical community has pursued angiogenesis as both a basic biological question and a therapeutic target. Decades later, it is striking how much we now know—and how much remains elusive. We have learned to block VEGF signaling in cancer and ophthalmic disease, yet resistance and incomplete efficacy remind us that angiogenesis is not governed by a single switch [8,9,10,11]. We have discovered that endothelial cells respond not only to growth factors but also to immune signals, metabolic conditions, and the mechanical properties of their environment [12]. They sit at the intersection of biology’s most fundamental forces, which is precisely why angiogenesis research never loses momentum [13,14,15].

This Special Issue particularly excels at capturing the full spectrum of that complexity, with research ranging from finely detailed molecular studies to broader clinical narratives, and from computational modeling to translational insights [16,17,18,19,20,21,22,23]. Consider, for example, how the VEGF/VEGFR system continues to challenge researchers. The relationship between ligands and receptors is not a tidy one; rather, it involves homo- and heterodimeric receptor activation, with context-dependent outcomes that make selective therapeutic targeting remarkably difficult. Several contributions in this collection revisit this problem, showing that even after decades of study, the VEGF family continues to surprise us [18,23,24]. Other articles shift the spotlight to immune–vascular crosstalk, an area where both editors themselves have made significant contributions [24]. VEGF family members do not simply promote vessel growth; they engage immune cells by sometimes activating them, and sometimes suppressing their functions. The review in this Issue traces these interactions with care, offering new frameworks for understanding why VEGF-targeted therapies sometimes show unexpected effects in immunotherapy [24]. The notion that macrophages can serve as architects [23] of new vasculature—by releasing VEGF-A during inflammation and tissue repair—adds another dimension to our understanding. These findings are not just mechanistic curiosities; they suggest that modulating macrophage polarization might open up new avenues for treating conditions such as peripheral artery disease.

The disease contexts explored in this Issue are as varied as they are clinically urgent. Systemic sclerosis, for instance, offers a striking example of what happens when angiogenesis fails. Here, endothelial dysfunction and endothelial-to-mesenchymal transition give rise to a paradoxical situation: excessive vascular injury coupled with defective vascular repair [19]. Similarly, in hypertensive kidney disease, changes in capillary density illustrate how the angiogenic machinery adapts—or maladapts—to chronic stress. These insights remind us that angiogenesis is never a generic process; it is tailored to the organ and to the disease microenvironment [20].

The papers in this collection also venture into fields that stretch traditional biological approaches. One study uses meshless computational modeling to explore how physical compression influences VEGF diffusion and capillary network development. Such work is emblematic of the future: angiogenesis is understood not only as a biochemical cascade but also as a biophysical and spatial phenomenon, sensitive to tissue mechanics and architecture [17]. In the same vein, contributions on retinopathy of prematurity show how developmental and metabolic cues shape pathological angiogenesis in the eye, again highlighting that angiogenesis is not one-size-fits-all but profoundly contextual [21].

Cardiovascular disease and cancer, two of the great burdens of our time, are of course never far from discussion [25,26]. In ischemic heart disease, VEGF is at once a potential biomarker and a therapeutic candidate. Its levels correlate with outcomes in heart failure and collateral vessel formation, yet its therapeutic use has proven challenging. Tumor angiogenesis, by contrast, embodies the darker side of the process: chaotic, relentless vessel growth that nourishes malignant cells [27]. The contributions in this Special Issue examine these aspects not as isolated curiosities but as points along a shared vascular continuum [18].

What strikes the reader when moving through this collection is how much the field has matured without losing its dynamism. Angiogenesis research is no longer confined to cataloging growth factors or mapping pathways; it is focused on integrating biology with physics, immunology, and clinical practice, and understanding how vessels behave across the three dimensions of being in living tissues, under stress, and in disease.

It is worth pausing to reflect on why angiogenesis continues to matter so profoundly. At a basic level, every tissue relies on vessels; angiogenesis is the infrastructure of life itself. But at a translational level, angiogenesis represents one of the few biological processes that is truly modifiable. We can block it, we can stimulate it, and—at least in principle—we can redirect it.

How do we heal ischemic tissues without feeding tumors? How do we promote vascular growth in the eye without losing vision? How do we harness macrophages to repair vessels without worsening inflammation? These are not abstract puzzles but matters of real clinical consequence.

In this sense, the Special Issue is more than a snapshot of current science; it is a roadmap charting where we have come from—decades of work on VEGF and angiogenic factors—and pointing toward where we need to go: therapies that are context-sensitive, patient-specific, and interdisciplinary in design. It reminds us that angiogenesis is not merely a biological process but a therapeutic opportunity, a lens through which to view both pathology and repair; invites us to think across boundaries, connecting cell biology with immunology, biophysics with pathology, and preclinical models with clinical trials; and invites us to continue the conversation about how best to guide angiogenesis—when to nurture it, when to restrain it, and how to predict its outcomes.

As angiogenesis continues to shape the future of biomedicine, this Special Issue stands as a catalyst demonstrating how far we have come in unraveling the mysteries of vascular growth, and how much promise remains in the journey ahead.
